# Cereus savings—successful use of dalbavancin in an immunocompromised patient with *Bacillus cereus* bacteremia

**DOI:** 10.1128/asmcr.00024-26

**Published:** 2026-09-09

**Authors:** Daisy L. Eaton, Matthew Stack, Robin Chamberland, Hunter O. Rondeau

**Affiliations:** 1 Department of Pharmacy, SSM Health DePaul Hospital 540819 , Bridgeton, Missouri, USA; 2 Division of Infectious Diseases, Allergy and Immunology, SSM Health Saint Louis University Hospital 25213, St. Louis, Missouri, USA; 3 Department of Pathology, SSM Health, Saint Louis University 7547, St. Louis, Missouri, USA; Pattern Bioscience, Austin, Texas, USA

**Keywords:** dalbavancin, *Bacillus cereus*, OPAT, immunocompromised, case report

## Abstract

**Background:**

Dalbavancin’s pharmacokinetics (long half-life, high tissue penetration, once-weekly dosing) make it an attractive alternative for patients with barriers to outpatient parenteral antimicrobial therapy or at risk for line-related complications. In this case, we used dalbavancin to promote timely discharge for an immunocompromised patient who experienced *Bacillus cereus* bacteremia due to a peripherally inserted central catheter (PICC) line-associated infection. Although *B. cereus* is known to cause line-related infections, we present a novel clinical case highlighting the successful use of dalbavancin in an immunocompromised patient with *B. cereus* bacteremia, and this approach also facilitated earlier discharge.

**Case Summary:**

A 58-year-old woman with invasive ductal breast cancer on chemotherapy (docetaxel, carboplatin, trastuzumab, pertuzumab), mechanical aortic valve replacement, and other comorbidities presented with febrile neutropenia 2 weeks after her fifth chemotherapy cycle. A month prior, she had completed vancomycin for a suspected port infection. Admission laboratory values revealed pancytopenia (white blood cell count [WBC] 1.7 × 10⁹/L, absolute neutrophil count [ANC] 0.53 × 10⁹/L, hemoglobin 7.8 g/dL, and platelets 51 × 10⁹/L). Both sets of blood cultures obtained prior to empiric vancomycin grew *B. cereus*, with positive cultures throughout day 3 despite treatment. A transthoracic and transesophageal echocardiography excluded endocarditis. The PICC line was removed on day 2; blood cultures cleared by day 5, and neutropenia resolved on day 7. Given two prior catheter-related infections and logistical challenges with weekend discharge planning, the patient received a single 1,500 mg dose of intravenous dalbavancin rather than receiving a new line and continuing prolonged intravenous therapy. This facilitated discharge 4 days earlier than anticipated. At 2.5-week follow-up and visits at 30, 60, and 90 days, the patient remained clinically well without recurrence of infection.

**Conclusion:**

This case highlights successful off-label use of dalbavancin in a complex case, suggesting that its use in such cases warrants further studies, especially when standard regimens can be logistically challenging.

## INTRODUCTION

*Bacillus cereus* is an environmental, gram-positive, spore-forming organism recognized as an opportunistic cause of invasive infection in immunocompromised hosts, particularly catheter-related bloodstream infections. Vancomycin is often the antimicrobial of choice due to its established clinical breakpoints that facilitate treatment decisions (1, 2). Dependence on central venous access can increase the risk of line-related complications and delay hospital discharge, especially in patients with barriers to outpatient parenteral antimicrobial therapy (OPAT). Dalbavancin, a long-acting lipoglycopeptide with potent gram-positive activity and extended half-life, has emerged as an off-label option for infections requiring prolonged treatment when traditional OPAT is unfeasible (3–6). Data supporting its use for *B. cereus* bacteremia, particularly in immunocompromised patients, remain limited. We report a case of *B. cereus* bacteremia in a neutropenic patient successfully managed with dalbavancin following line removal, highlighting its potential to facilitate early discharge and avoid prolonged intravenous access.

## CASE PRESENTATION

A 58-year-old woman with a medical history most notable for invasive ductal breast cancer (on chemotherapy), type II diabetes, hypertension, aortic valve stenosis (status post aortic mechanical valve replacement in 9/2019, on warfarin), chronic obstructive pulmonary disease, obstructive sleep apnea, obesity (status post gastric bypass in 2017), and hospitalization about a month prior due to suspected infection of her right chemo chest port (status post 7 days of intravenous vancomycin from the date of port removal) was directly admitted to our hospital from her oncologist’s office due to concern for sepsis or possible urinary tract infection. The patient endorsed feeling poorly overall since her fifth cycle of chemotherapy, which was about 2 weeks prior to her current admission. The patient’s chemotherapy regimen was docetaxel, carboplatin, trastuzumab, and pertuzumab every 3 weeks. The patient stated that she typically felt ill for a few days after chemotherapy and then recovered. However, she never improved. Her worsening symptoms prompted a visit with her oncologist. She endorsed generalized malaise, nausea, lower abdominal cramping, diarrhea, dysuria, and subjective fevers and chills. As a result, her oncologist instructed her to present to the emergency room for direct admission.

**Fig 1:**
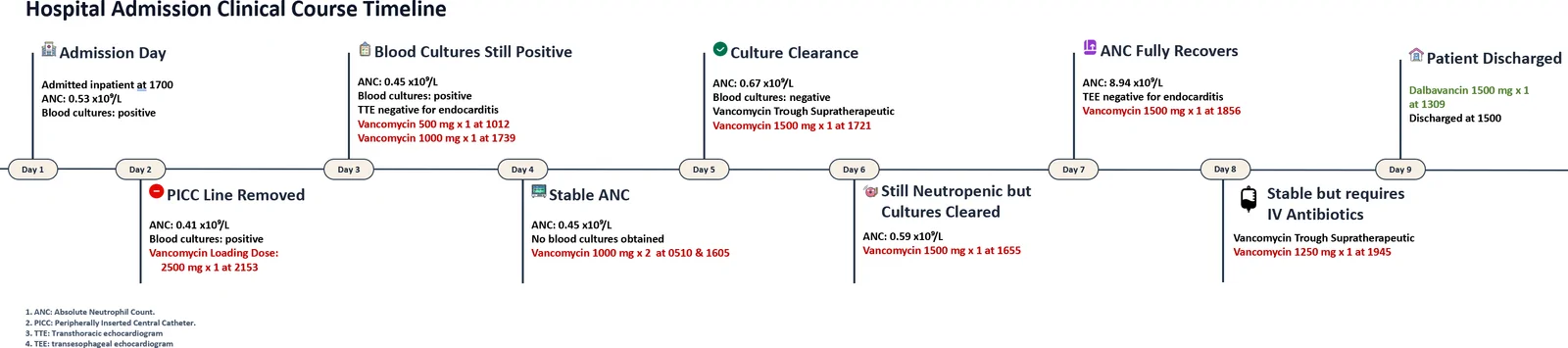
Hospital admission clinical course timeline. Clinical course from admission to discharge, including serial ANC values, blood culture results, echocardiography, PICC removal, and antimicrobial therapy. ANC, absolute neutrophil count; PICC, peripherally inserted central catheter; TTE, transthoracic echocardiogram; TEE, transesophageal echocardiogram.

A urinalysis obtained upon admission was negative. Her labs on the day of admission were notable for a white blood cell count of 1.7 × 10⁹/L (reference 4.0–10.7), absolute neutrophil count of 0.53 × 10⁹/L (reference 1.60–7.50), hemoglobin of 7.8 g/dL (reference 11.9–15.8), and platelet count of 51 × 10⁹/L (reference 150–420). Both sets of peripheral blood cultures obtained on the day of admission, before initiation of empiric antibiotic therapy, grew *B. cereus,* identified by matrix-assisted laser desorption/ionization time-of-flight mass spectrometry (MALDI-TOF MS, Vitek MS Prime, version 3.2, Library version 9.04). After ruling out *Bacillus anthracis* based on β-hemolysis, identification of *B. cereus* was confirmed by MALDI and phenotypic methods, and susceptibility testing was performed by a reference laboratory using the broth microdilution method with custom-made MIC panels, and the results were interpreted according to CLSI M45 ED3 (Table 1) (1). The patient started intravenous vancomycin. Follow-up blood cultures on days 2 and 3 were positive for *B. cereus*, but blood cultures cleared by day 5 of admission. Given the presence of the mechanical aortic valve and persistently positive blood cultures, the patient underwent a transthoracic echocardiogram (TTE) and transesophageal echocardiogram (TEE) to evaluate for possible infective endocarditis; these showed no evidence of endocarditis. Meanwhile, the peripherally inserted central catheter (PICC) line was removed on day 2 of admission, and neutropenia resolved on day 7 of admission (see Fig. 1 for timeline of events). As a result, the most likely source of the *B. cereus* bacteremia was considered to be the peripherally inserted central catheter (PICC) line, especially since blood cultures cleared after PICC line removal. Of note, the PICC line was in place for 3 weeks when she initially presented from her oncologist’s office.

**TABLE 1:** *B. cereus* susceptibilities^*a*^

Drug	MIC (µg/mL) for *B. cereus*	Interpretation
Penicillin	≥16	Resistant
Meropenem	0.06	Susceptible
Ceftriaxone	≥4	N/A^*b*^
Vancomycin	1	Susceptible
Gentamicin	≤2	Susceptible

^*a*^ From patient’s initial blood cultures.

^*b*^ N/A, not applicable, as there are no established breakpoints for ceftriaxone and *B. cereus*.

Given that the patient felt better, blood cultures cleared, endocarditis was ruled out, and PICC line removal was completed, preparations were made for discharge. Initially, the plan was to insert another PICC line or midline and discharge the patient on a course of i.v. vancomycin to complete therapy for the line-related infection. However, it was unclear if the patient could get another PICC line or midline placed and obtain insurance approval for i.v. vancomycin with home health before the weekend. If not, the patient would remain admitted over the weekend. Given the timing constraints and the patient’s two prior central line-related infections, a decision was made to give one dose of i.v. dalbavancin 1500 mg and discharge the patient home. The patient had been on 7 days of i.v. vancomycin when she was transitioned to dalbavancin therapy. This obviated the need for a PICC or midline, home health, and home i.v. antibiotics. Additionally, use of dalbavancin facilitated the patient being discharged about 4 days earlier than initially anticipated.

The patient was discharged in a timely fashion before the weekend and followed up in the Infectious Disease Clinic about 2.5 weeks later. The patient reported doing well overall. She denied any fevers, chills, or other systemic symptoms. Earlier discharge, avoidance of PICC-line placement and maintenance, and eliminating the need for home i.v. antibiotics were significant contributors to patient satisfaction. The patient continued to do well overall, without evidence of recurring infection at 30, 60, or 90 days of follow-up.

## DISCUSSION

*B. cereus* is an aerobic, gram-positive, spore-forming bacillus that, along with *B. anthracis* and several other species, makes up the *B. cereus* group. *B. cereus* is found in the environment and can act as an opportunistic pathogen, causing a variety of infections. Laboratory differentiation of members of the group can be challenging, and care must be taken to rule out *B. anthracis* (often not included in identification databases) before additional testing; in this case, this was based on the presence of β-hemolysis. *B. cereus* exhibits resistance to penicillins and cephalosporins because of beta-lactamase production; thus, vancomycin, clindamycin, carbapenems, or fluoroquinolones are preferred therapeutic agents pending susceptibility results (2). Susceptibility testing for *Bacillus* species is not offered on commercial devices and is thus not readily available in many laboratories. CLSI interpretive guidelines are available for broth microdilution (BMD) testing, as well as European Committee on Antimicrobial Susceptibility Testing (EUCAST) guidelines for both BMD and disk diffusion.

Dalbavancin is a long-acting lipoglycopeptide with potent activity against the following gram-positive organisms: *Staphylococcus aureus, Streptococcus pyogenes, Streptococcus agalactiae, Streptococcus dysgalactiae, Streptococcus anginosus* group, and *Enterococcus faecalis* (3). Susceptibility testing interpretive criteria exist for dalbavancin for these organisms, but not for *B. cereus*. Vancomycin can reliably predict susceptibility to dalbavancin in *S. aureus* and β-hemolytic streptococci, but this has not been demonstrated in *Bacillus* species. While dalbavancin is U.S. Food and Drug Administration (FDA) approved for acute bacterial skin and skin structure infections, there is off-label use for infections where long durations of therapy are indicated (3). Its most established off-label use is step-down therapy for treating endocarditis in persons who inject drugs (PWID). Its favorable pharmacokinetic profile, characterized by a long half-life of approximately 14.5 days, high tissue penetration, and once-weekly dosing, shows potential benefits for patients with barriers to OPAT (4).

Several studies demonstrated dalbavancin’s utility in complex infections and vulnerable populations. Tuan et al. reported a 93% clinical cure rate in patients with diverse infections, including bacteremia, endocarditis, and osteomyelitis, with many ineligible for OPAT due to unstable housing, substance use, or lack of support (5). Similarly, Zambrano et al. found comparable cure rates between PWID and non-PWID, despite higher rates of patient-directed discharge and loss to follow-up in the former group. These findings underscore dalbavancin’s role in facilitating treatment completion in populations traditionally considered high-risk for nonadherence (6).

The DOTS randomized clinical trial provided evidence for dalbavancin’s non-inferiority to standard-of-care antibiotics in treating *S. aureus* bacteremia (7). Patients receiving dalbavancin had similar clinical success rates and fewer catheter-related complications, supporting its use as a safe and effective alternative to prolonged i.v. therapy. Although *B. cereus* is not a common pathogen in these studies, its gram-positive nature and susceptibility to glycopeptides suggest that dalbavancin may be a viable option in select cases, especially when source control and clinical stability are achieved. In this patient’s case, she already previously had two central line infections; thus, dalbavancin obviated the need for another central line and possibly another central line infection. Bao et al. further supported dalbavancin’s role in treating deep-seated infections such as osteomyelitis and hardware-associated infections, noting its efficacy and safety in patients with difficult venous access or vancomycin intolerance (8).

Our patient’s immunocompromised status added complexity to antimicrobial decisions. Immunosuppressed individuals often require prolonged therapy and are at increased risk for complications (9). The ability to administer dalbavancin in the inpatient setting to allow for timely discharge reduced the risk of hospital-acquired infections and improved quality of life; this also prevented the need for an additional central line, as well as costs associated with home healthcare and home i.v. antibiotics. While data on dalbavancin use in immunocompromised hosts remain limited, favorable outcomes in real-world cohorts suggest that it may be a reasonable option when standard regimens are impractical or contraindicated (6). We do acknowledge limitations, including the lack of dalbavancin susceptibilities, the use of this therapy in a single patient, and PICC removal for source control, which limits generalizability to other populations.

Another advantage of providing our patient with a single dalbavancin dose compared to OPAT with vancomycin was cost reduction. A 500 mg dose of dalbavancin costs approximately $2,134 (10). While vancomycin costs less per dose, it also requires frequent testing to ensure that adequate levels are achieved and requires healthcare providers to administer it either inpatient or through home healthcare services. It is estimated that, in our state, each day of hospital admission costs patients around $2,803 (11). Based on this, our patient’s net hospital costs were reduced by approximately $4,810 by using a single 1,500 mg dose of dalbavancin and reducing length of stay by 4 days.

In conclusion, this case highlights the successful use of dalbavancin in an immunocompromised patient with *B. cereus* bacteremia, where traditional inpatient intravenous therapy posed logistical and clinical challenges. Transitioning to dalbavancin enabled early discharge and completion of antimicrobial therapy and prevented further treatment complications. This case also contributes to the growing body of evidence supporting dalbavancin as a patient-centered antimicrobial agent and highlights the need for research into its efficacy against pathogens such as *B. cereus*, especially in immunocompromised populations. Future studies should explore microbiologic outcomes, optimal dosing strategies, long-term safety, and cost savings to further establish dalbavancin’s role in infections caused by less common gram-positive organisms.
